# Emergence of rifampicin-resistant staphylococci on the skin and nose of rifampicin-treated patients with an orthopaedic-device-related infection

**DOI:** 10.5194/jbji-9-191-2024

**Published:** 2024-07-24

**Authors:** Alexandra Wallimann, Yvonne Achermann, Ciara Ferris, Mario Morgenstern, Martin Clauss, Vincent Stadelmann, Hannes Andreas Rüdiger, Liam O'Mahony, Thomas Fintan Moriarty

**Affiliations:** 1 AO Research Institute Davos, Davos, Switzerland; 2 Swiss Institute of Allergy and Asthma Research (SIAF), University of Zurich, Davos, Switzerland; 3 Department of Internal Medicine, Hospital Zollikerberg, Zollikerberg, Switzerland; 4 Department of Dermatology, University Hospital Zurich, Zurich, Switzerland; 5 Department of Infectious Diseases and Hospital Epidemiology, University of Zurich, Zurich, Switzerland; 6 Center for Musculoskeletal Infections (ZMSI), University Hospital Basel, Basel, Switzerland; 7 Department of Orthopedic and Trauma Surgery, University Hospital Basel, Basel, Switzerland; 8 Department of Research and Development, Schulthess Klinik, Zurich, Switzerland; 9 Department of Hip and Knee Surgery, Schulthess Klinik, Zurich, Switzerland; 10 Departments of Medicine and Microbiology, APC Microbiome Ireland, University College Cork, College Road, Cork, Ireland

## Abstract

Rifampicin is a key antibiotic in the treatment of staphylococcal biofilm infections. In this pilot study, we found that patients who received rifampicin for treatment of an orthopaedic-device-related infection (ODRI) were colonized with rifampicin-resistant staphylococci during treatment and this persisted for up to 2 months after cessation of treatment.

## 1 Introduction

Due to the importance of biofilms in orthopaedic-device-related infection (ODRI), antimicrobial agents with anti-biofilm activity are the key components of treatment algorithms. Skin commensal bacteria such as *Staphylococcus aureus* or coagulase-negative staphylococci are often the source of infection in ODRIs (Masters et al., 2022). Rifampicin is a widely used agent in the treatment of staphylococcal biofilm infections due to its efficacy against staphylococci in biofilms (Zimmerli and Sendi, 2019). Rifampicin binds to the β subunit of the bacterial RNA polymerase, thereby inhibiting bacterial transcription. Single-nucleotide mutations in the β -subunit-encoding *rpoB* gene result in amino acid substitution causing reduced affinity to rifampicin (Wi et al., 2018; Zimmerli and Sendi, 2019). This mutation occurs readily in both *S. aureus* and coagulase-negative staphylococci exposed to rifampicin under permissive conditions (e.g. monotherapy) (Achermann et al., 2013; Goldstein, 2014; Wi et al., 2018). The management of a rifampicin-resistant staphylococcal ODRI is challenging (Zimmerli et al., 1998). Achermann et al. (2013) showed that (inadequate) rifampicin treatment is an independent significant risk factor for emergence of rifampicin resistance in the infecting pathogen. Rifampicin therapy is generally delayed until surgical wounds are dry and healing to reduce the risk of superinfection with rifampicin-resistant staphylococci that may emerge, for example, on the skin of treated patients. There is, however, no clinical data on how fast rifampicin-resistant staphylococci emerge on the skin and how long they persist during and after rifampicin treatment. The aim of this study was to investigate whether rifampicin-resistant staphylococci emerge on the skin or in the nose of ODRI patients undergoing antibiotic therapy with rifampicin compared to controls receiving other antibiotics.

## 2 Material and methods

### 2.1 Patient recruitment

We prospectively enrolled patients from two Swiss orthopaedic hospitals (Schulthess Klinik, Zurich, and the University Hospital Basel) from January 2020 until end of January 2022. Patient samples were taken within the context of a clinical study looking at the effect of antibiotic therapy on the gut microbiota.

Subjects were eligible if they were at least 18 years old and scheduled to undergo revision surgery due to a confirmed ODRI (hip or knee periprosthetic joint infection or fracture-related infection). Subjects were excluded from the study if they took more than one dose of antibiotics in the 6 weeks prior to recruitment, if they suffered from gut-associated morbidities, if the antibiotic therapy had already started prior to the baseline sampling, or if they were unable to give consent and follow procedures due to insufficient knowledge of the project language or due to psychiatric disorders. Patients were given 24 h to consider joining the study before giving written consent. An overview of the recruited patients ( n=12 ), including the pathogens causing the infections and the prescribed antibiotic therapy, is provided in Table 1. This multicenter study was approved by the cantonal ethical commission of Zurich (2019_00635) and is registered at https://clinicaltrials.gov/ (last access: 3 February 2023) (NCT04440631).

**Table 1:** Basic characteristics, infecting pathogen and antibiotic treatment in 12 patients with an orthopaedic-device-related infection.

Patient	Gender, age	Pathogen	Antibiotic therapy	Duration
	[years]			[d]
162	Female, 57	*Cutibacterium avidum*	2.2 g 4× per day amoxicillin/clavulanic acid IV	6
			0.4 g 6× per day penicillin IV	3
			300 mg 3× per day clindamycin oral	34
185	Female, 79	*Enterococcus faecalis*, penicillin-susceptible	2.2 g 3× per day amoxicillin/clavulanic acid IV	37
223	Female, 66	*Staphylococcus aureus*, methicillin-susceptible	2.2 g 3× per day amoxicillin/clavulanic acid IV	6
			500 mg 3× per day daptomycin IV	1
344	Female, 72	*Pseudomonas aeruginosa*	2 g 3× per day meropenem oral	10
			750 mg 2× per day ciprofloxacin oral	78
646	Male, 41	*Staphylococcus aureus*, methicillin-susceptible	2.2 g 3× per day amoxicillin/clavulanic acid IV	8
			960 mg 3× per day cotrimoxazole oral	15
647	Male, 72	*Streptococcus agalactiae*	2.2 g 4× per day amoxicillin/clavulanic acid oral	5
			5 Mio IE* 4× per day penicillin G IV	35
			2.2 g 4× per day amoxicillin/clavulanic acid IV	8
567	Male, 77	*Finegoldia magna*, *Staphylococcus epidermidis*, *Cutibacterium acnes *(polymicrobial)	2.2 g 3× per day amoxicillin/clavulanic acid IV	8
			400 mg 1× per day moxifloxacin oral	36
287	Female, 80	*Staphylococcusepidermidis*,methicillin-resistant	2.2 g 3× per day amoxicillin/clavulanic acid IV	7
			1 g 2× per day vancomycin IV	9
			960 mg 2× per day cotrimoxazole oral	74
			450 mg 2× per day rifampicin oral	76
753	Female, 55	*Staphylococcus epidermidis*, methicillin-resistant	750–1250 mg 2× per day vancomycin IV	8
			960 mg 3× per day cotrimoxazole oral	2
			500 mg 2× per day levofloxacin oral	76
			450 mg 2× per day rifampicin oral	79
888	Female, 80	*Staphylococcus epidermidis*,methicillin-resistant	1 g 2× per day vancomycin IV	7
			600 mg 2× per day linezolid oral	28
			500 mg 3× per day fucidin acid	56
			300 mg 2× per day rifampicin oral	84
928	Male, 65	*Staphylococcus epidermidis*,methicillin-resistant	2.2 g 3× per day amoxicillin/clavulanic acid IV	8
			2 g 4× per day flucloxacillin IV	3
			500 mg 2× per day levofloxacin oral	80
			450 mg 2× per day rifampicin oral	80
966	Male, 60	*Staphylococcus aureus*,rifampicin-resistant	3× 1 g per day; 4×2 g per day amoxicillin/clavulanic acid oral	3
			2 g d^-1^ cefazolin IV	23
			450 mg 2× per day rifampicin oral	5

* Mio IE: million international units.

### 2.2 Study procedure

Samples from all included patients were taken at four different time points: at baseline (prior to initiation of antibiotic therapy); during intravenous antibiotic therapy (2 weeks after antibiotic therapy started); during oral antibiotic therapy, which includes rifampicin for five of the patients (6 weeks after antibiotic therapy started); and after a 24-week follow-up, at which time patients were off antibiotics for a minimum of 2 months.

### 2.3 Skin and nose swab collection

To determine the presence of rifampicin-resistant staphylococci on the skin and nose of the patients, swabs were collected using the Copan eSwab^®^ (80490CEA) system. In brief, a skin swab was taken in the cubital fossa of the right arm by gently rubbing 30 times back and forth. The nose swab was taken by placing the swab approximately 0.5 cm into the right nostril and by turning the swab gently 10 times. Swabs were placed back in the tube containing the liquid Amies medium and sent to the laboratory in Davos at ambient temperature. Samples were plated as early as possible upon arrival and leftover liquid was frozen at - 80 °C to perform DNA isolation at the end of the recruitment period, when all samples were collected. The skin swab collection and analysis were not part of the diagnostic routine for these patients. Identification of rifampicin-resistant staphylococci was not reported to the treating physician.

### 2.4 Determination of rifampicin resistance 

For the analysis of potential rifampicin-resistant staphylococci on the skin and nose of the patients, 100 µL of the liquid Amies medium was plated both on pure mannitol salt agar (MSA) plates (CM0085; Oxoid) and MSA plates containing 1 µg mL^-1^ rifampicin. Plates were checked for colonies after a 24 h incubation at 37 °C. *S. aureus* (CCOS 890), *S. epidermidis* (ATCC 35984) and a methicillin-and-rifampicin-resistant *S. aureus* (EDCC 5443) were used as controls. Any colonies growing on the rifampicin-containing plate were frozen away in 20 % glycerol in Mueller–Hinton broth (MHB).

Isolates were further investigated for rifampicin resistance by a zone of inhibition (ZOI) assay. Isolates growing on the rifampicin-containing MSA plates were tested for rifampicin resistance by streaking out on Mueller–Hinton agar (MHA) plates according to EUCAST SOP 9.3, and three antimicrobial susceptibility discs containing 5 µg of rifampicin were placed on the plate. ZOI was assessed after a 24 h incubation period at 37° by means of a Scan 1200 inhibition zone reader (Interscience).

By means of MALDI-TOF (Synlab Int GmbH, Lucerne, Switzerland), species of rifampicin-resistant candidates were identified.

In a final step, DNA from all the rifampicin-resistant isolates was extracted using the QIAmp mini kit (Qiagen). Prior to the DNA purification, which was performed according to manufacturer's instructions, a lysis step with 50 µg mL^-1^ lysostaphin and 1 mg mL^-1^ lysozyme was performed along with a proteinase K digestion of the sample.

To detect potential nucleotide mutations, polymerase chain reaction (PCR) of the *rpoB* gene was performed. Platinium^®^ Superfi™ II PCR master mix (Invitrogen^®^), primer rpoBfor (GTCGTTTACGTTCTGTAGGTG) and primer rpoBrev (TCAACTTTACGATATGGTGTTTC) were used. PCR amplification conditions were 94 °C for 9 min 30 s (initial denaturation step), 94 °C for 30 s, 62 °C for 30 s and 72 °C for 1 min for 35 cycles, and 72 °C for 10 min (final extension step). Primers and PCR amplification conditions were previously described by Mick et al. (2010). The PCR product was purified from a 1 % agarose gel by means of the PureLink^®^ PCR purification kit (Invitrogen™). Purified PCR products were sent for Sanger sequencing at Microsynth AG (Balgach, Switzerland). Obtained sequences from clinical isolates were compared to reference strains *S. aureus* (GenBank accession no. CP089586.1), *S. haemolyticus* (GenBank accession no. CP035291.1) and *S. epidermidis* (GenBank accession no. CP043845.1).

## 3 Results

We included 12 patients (7 females and 5 males) with a median age of 69 years (range 41–80 years). They had either a hip periprosthetic joint infection (PJI; n=10 ) or a fracture-related infection (FRI; n=2 ) caused by *S. aureus* ( n=3 ), by coagulase-negative staphylococci ( n=4 ) or by another bacterial pathogen ( n=5 ) (Table 1). After surgical debridement of infected tissue, 5 out of 12 patients received oral rifampicin in combination with other antibiotic agent(s), while 7 received antibiotic treatment excluding rifampicin. Since rifampicin-resistant staphylococci were found during the surgical debridement in one patient (patient 966), rifampicin treatment was stopped after 5 d. The median duration of rifampicin treatment was 79 d (range of 5–84 d). We missed our initial recruitment goal of 80 patients mainly due to the Covid pandemic, which made the enrolment of study patients difficult, and, as this study was conducted within the context of a gut microbiota study requiring faecal sampling prior to receiving antibiotic therapy, many patients were not eligible for inclusion as they had already started antibiotic therapy due to severe infection.

Out of 12 patients, two were colonized with a rifampicin-resistant strain at baseline (Table 2). During treatment, no taken nasal swab detected a rifampicin-resistant strain at 2 weeks, but at 6 weeks, three patients were colonized with a rifampicin-resistant strain (one in the non-rifampicin group and two in the rifampicin group). At 24 weeks (about 8 weeks after treatment stop), 5 out of 12 patients were colonized with a rifampicin-resistant strain – namely all of the rifampicin treatment group.

**Table 2:** A timeline of rifampicin-resistant staphylococci in the nose and on the skin, with detailed information about mutations in *rpoB* genes.

**(a)** Microbial analysis of patients receiving rifampicin									
		Baseline		2 weeks		6 weeks		24 weeks	
Patient	Pathogen	Nose	Skin	Nose	Skin	Nose	Skin	Nose	Skin
287	*S. epidermidis*	Negative	Negative	Negative	Negative	Negative	Negative	*S. epidermidis* ^1^	*S. epidermidis* ^1^
753	*S. epidermidis*	Negative	Negative	Negative	Negative	S. *aureus* No mutationdetected	*S. aureus* No mutationdetected	S. *epidermidis* ^2,3,4^	*S aureus* No mutationdetected
888	*S. epidermidis*	*S. epidermidis* ^1^ *S. haemolyticus* ^1^	S. *epidermidis*^1^*S. haemolyticus*Ambiguous nucleotides	Negative	*S. epidermidis*^1^*S. haemolyticus* Ambiguous nucleotides	No growth	No growth	*S. epidermidis* ^3,5^	*S. epidermidis* ^1^
928	*S. epidermidis*	Negative	Negative	Negative	Negative	*S. epidermidis* ^6^	No growth	*S. epidermidis* ^7^	*S. epidermidis* ^7^
966	*S. aureus* (rifampicin-resistant)	Negative	Negative	Negative	No growth	No growth	No growth	S. *haemolyticus* Ambiguous nucleotides	*S. haemolyticus* Ambiguousnucleotides
**(b)** Microbial analysis of patients without rifampicin									
		Baseline		2 weeks		6 weeks		24 weeks	
Patient	Pathogen	Nose	Skin	Nose	Skin	Nose	Skin	Nose	Skin
162	*C. avidum*	Negative	Negative	Negative	Negative	Negative	Negative	Negative	Negative
185	*E. faecalis*	Negative	Negative	No sample	No sample	Negative	Negative	Negative	Negative
223	*S. aureus*	Negative	No growth	No growth	No growth	Negative	No growth	No sample	No sample
344	*P. aeruginosa*	Negative	Negative	Negative	No growth	Negative	Negative	Negative	Negative
646	*S. aureus*	Negative	No growth	No sample	No sample	Negative	No growth	Negative	Negative
647	*S. agalactiae*	Negative	Negative	No sample	No sample	Negative	Negative	Negative	Negative
567	*Polymicrobial*(*F. magna*, S. *epidermidis*, *C. acnes*)	*S. haemolyticus* ^8,9^	Negative	No sample	No sample	S. *haemolyticus* ^8,9^	S. *haemolyticus* ^8,9^	Negative	Negative

^1^ Asp (gac) → Glu (gaa) (46). ^2^ Asp (GAC) → Glu (GAA) (38). ^3^ Ile (ATA) → Met (ATG) (102). ^4^ Cys (TGT) → (T–T) (104); deletion – shift in reading frame. ^5^ Asp (GAC) → Glu (GAA) (48). ^6^ Ala (GCA) → Glu (GAA) (52). ^7^ Ser (TCT) → Phe (TTT) (61). ^8^ Asp (GAC) → Glu (GAG) (38). ^9^ Ile (ATT) → Met (ATG) (94). Rif = rifampicin, Negative: no growth of rifampicin-resistant staphylococci, no sample: no sample received, no growth: no growth of any bacteria on antibiotic-free MSA. Silent mutations not mentioned. Abbreviations – *S. epidermidis*: *Staphylococcus epidermidis*, *S. aureus*: *Staphylococcus aureus*, *C. avidum*: *Cutibacterium avidum*, *E. faecalis*: *Enterococcus faecalis*, *P. aeruginosa*: *Pseudomonas aeruginosa*, *S. agalactiae*: *Streptococcus agalactiae*, *F. magna*: *Finegoldia magna*, and *C. acnes*: *Cutibacterium acnes*.

A similar result was seen in samples taken from the skin. Colonization with a rifampicin-resistant strain was seen in two patients at 2 weeks, in one patient at 6 weeks and in five patients at 24 weeks.

The rifampicin-resistant isolates within the treatment and follow-up were found to be *S. epidermidis* ( n=11 ), *S. haemolyticus* ( n=7 ) or *S. aureus* ( n=3 ) (Table 2). All the presumed rifampicin-resistant isolates grew on MHA, whereby the ZOI was not detectable (0 mm ZOI), which is defined as high-level resistance (Goldstein, 2014). The mutations in the *rpoB* gene responsible for rifampicin resistance were all single mutations and are shown in Table 2. Out of the five rifampicin-resistant *S. haemolyticus* strains, four showed ambiguous nucleotide mutations, but all have the potential to cause rifampicin resistance.

## 4 Discussion

The therapy of ODRI often requires elaborate treatment including both surgical and antibiotic therapy for successful outcomes. A rifampicin-resistant staphylococcal infection can be challenging due to the importance of rifampicin in treating biofilm. Achermann et al. (2013) showed, in a retrospective study of patients with a rifampicin-resistant staphylococcal infection, that 85 % had a previous rifampicin therapy. In the current study, we detect rifampicin-resistant staphylococci on the skin or in the nose in all five included patients taking rifampicin for the diagnosed PJI. The detection of rifampicin-resistant staphylococci persisted for at least 2 months after stopping the antibiotic therapy. Other studies have shown that rifampicin-resistant methicillin-resistant *S. aureus* (MRSA) first appeared at a median of 9 d (Lai et al., 2010) or 12 d (Ju et al., 2006) after rifampicin-containing antibiotic therapy started in the blood and wound respectively; however, it is not described if the rifampicin resistance persisted in these cohorts. The prolonged skin and nasal colonizations highlight the recommendation that rifampicin treatment should be limited and only be given by specialists in orthopaedic infections when criteria were fulfilled (staphylococcal infection with a retained prosthesis, dry wound, well-controlled infection, knowledge about a potent combination partner with rifampicin and no relevant rifampicin interaction) (Achermann et al., 2013). Potentially, should these patients undergo further surgery within 6 months after antibiotic rifampicin treatment (e.g. revision), such patients may be at elevated risk for a second infection with a rifampicin-resistant staphylococcus.

We found various mutations in the bacterial genome with amino acid substitutions in this study; some of them have previously been described as being in correlation with rifampicin resistance in *S. epidermidis* (Hellmark et al., 2009; Padayachee and Klugman, 1999; Wi et al., 2018). It remains to be clarified if the detected mutations in the bacterial genome are transient or persist beyond the 24-week time point.

The low number of patients and differences in adherence regarding samples taken between the study groups and sampling sites limit the power of this study. Future studies are warranted based on our findings, with a larger sample size. Our pilot data may serve as a power calculation for future studies. In future studies, several swabs should be taken from different skin sites on the body and from the same region to overcome the challenge of heterogenous colonization and reduce sampling error. Importantly, the missing samples were primarily from the non-rifampicin group and at the 2-week time point. Therefore, these samples were prior to rifampicin exposure and do not influence the interpretation of resistance emerging during and after treatment.

In conclusion, we found that oral rifampicin therapy leads to consistent and persistent induction of resistance in commensal staphylococci on the skin and in the nose for a prolonged time. If patients get re-operated on within this window, physicians should be aware of these findings. Our results should be confirmed in larger patient cohorts with longer follow-up data to investigate for how long the rifampicin resistance persists and if the resistant clones are identical between the different sampling sites and times.

## Data Availability

Data used and analyzed are available upon reasonable request.
